# The future of pharmacy work: How pharmacists are adapting to and preparing for technology infusion

**DOI:** 10.1016/j.rcsop.2024.100472

**Published:** 2024-07-05

**Authors:** Nataly Martini, Laszlo Sajtos, Lynette Idio, Manvinder Kaur, Nicole Sweeney, Carrie Zhang, Shane Scahill

**Affiliations:** a School of Pharmacy, The University of Auckland, Auckland, New Zealand; b Department of Marketing, Business School, University of Auckland, Auckland, New Zealand

**Keywords:** Pharmacy, Future of work, Fourth industrial revolution, Technology, Automation, COVID-19

## Abstract

**Background:**

The pharmacy sector is rapidly evolving due to technological advancements, presenting challenges and opportunities for pharmacists. However, limited literature exists on the future of pharmacy work, especially concerning technology adoption.

**Objective:**

This exploratory study investigates pharmacists' perspectives on the impact of technologies on the profession - including career security, role evolution, adjustments to changes - and the impact of the COVID-19 pandemic on technology implementation and the broader future of work in pharmacy.

**Method:**

A cross-sectional survey design was used, targeting all registered pharmacists in New Zealand. A questionnaire, adapted from Future of Work literature, was piloted and distributed to 3037 pharmacists. Data were analyzed using descriptive statistics, two-step hierarchical analysis, and content and thematic analysis. Ethics approval was obtained.

**Results:**

177 responses met the inclusion criteria, yielding a 5.82% response rate. Respondent demographics included a lower proportion of community pharmacists and individuals of Asian ethnicity, but a higher proportion of males and hospital pharmacists compared to the national workforce. Most respondents were aged between 30 and 59 years, representing all District Health Board locations.

Qualitative analysis identified two themes: 1) Factors affecting technology adoption across macro, *meso* and micro levels, including COVID-19's impact on work efficiency, regulatory gaps, fragmented IT and organizational infrastructures, patient safety, and attitudes at workforce and individual levels; 2) Career impacts, highlighting role expansion, job replacement fears, and the need for adaptation and upskilling. Quantitative findings indicate that early technology adopters are more prepared to learn new skills and plan their careers. Technology impact positively correlates with career planning, while job loss concerns negatively affect skill development readiness.

**Conclusion:**

The study underscores the importance of early technological adoption for readiness to acquire new skills and career planning in pharmacy. Embracing technological change, supported by regulatory and policy frameworks, is crucial for advancing the profession.

## 1 Introduction

The rapidly evolving landscape of work is at the forefront of scholarly inquiry, industry practices, and policy-making discussions. The concept of the *‘future of work’* has emerged as a pivotal area of study, an important academic discipline that addresses the profound transformations in the nature of work (what we do), the workforce (who does it), and the workplace (where it's done).^1^ At the heart of this discourse is the exploration of how smart technology, automation, artificial intelligence (AI), robotics, and algorithms - together abbreviated as STAARA - are outperforming humans in many tasks,^2^^,^^3^ thereby transforming the work landscape^1^^,^^4^ and making these technologies increasingly attractive for employers.

This shift has profound implications for the delivery of healthcare, where tasks such as information gathering, analysis, and non-clinical decision-making are more susceptible to automation.^5^ However, the replication of complex human emotions and interpersonal interactions remain a formidable challenge to reproduce using technology,^6^^,^^7^ which is believed to safeguard roles that necessitate human communication, creativity and dexterity^8^, ^9^, ^10^ from the threat of unemployment.^6^^,^^8^^,^^9^

In the specific context of pharmacy, the sector stands at a crossroads of challenges and opportunities brought about by technological innovation^11^ highlight the evolving role of pharmacists from traditional duties towards more patient-centered care, a transition supported and facilitated by technological tools. From barcode scanning to sophisticated automated dispensing systems,^12^ technology is revolutionizing the pharmacy profession, improving auditing and storage of medication,^10^^,^^13^ reducing medication errors in hospital pharmacies,^14^^,^^15^ and enhancing workflow efficiency, patient care, and medication adherence.^16^

However, the integration of technology is not without its complexities, raising concerns about the overarching impact on the pharmacist's future of work and the viability of the profession into the longer term. Within New Zealand community pharmacy, a small pilot study indicates there is a sense of optimism regarding the potential of technology to enhance workflow efficiency, minimize errors, and streamline processes, thereby fostering an environment conducive to patient-centered care.^16^ However, whether technology truly frees up time for dispensary staff and increases patient interactions is debatable.^17^^,^^18^ Where some evidence suggests that automated dispensing systems can alleviate workload and reduce dispensing times,^19^, ^20^, ^21^ other reports indicate that automation may inadvertently prolong dispensing times without appreciably impacting patient counselling sessions or enhancing pharmacists' job satisfaction.^18^ Compounding these concerns are apprehensions regarding the need for extensive staff training,^3^ the administrative and troubleshooting burdens accompanying technology adoption,^3^^,^^16^ and the fear of potential job losses, programming errors, and an over-reliance on technology that inadvertently could lead to additional errors and increased workload.^7^^,^^16^ Furthermore, some pharmacists are anxious about an ‘industrial future’ where their expertise could be usurped by machines.^22^

This exploratory study aims to investigate the views of New Zealand pharmacists on how STAARA technologies are influencing the profession, their concerns regarding career security, the adoption of new technologies, and how pharmacists' roles might evolve with ongoing technological advancements, as well as what pharmacists are doing to adjust to these changes. Additionally, the study seeks to understand the effect of the COVID-19 pandemic on technology implementation and the broader future of work in the sector.

The rationale for this study is multifaceted; driven by the rapid technological evolution within healthcare and the contrasting evidence on its benefits and challenges. It is especially appropriate in the wake of the COVID-19 pandemic, which has accelerated technology adoption in healthcare, altering the dynamics of patient care and pharmacy practice.^23^ By examining pharmacists' perceptions and experiences, the study aims to fill a crucial knowledge gap, offering insights that inform policy, education, and practice enhancements. Addressing the opportunities and hurdles presented by STAARA, this research seeks to guide the pharmacy profession towards effective integration of technology innovation, ensuring both the sustainability of the profession and the enhancement of patient-centered care in an evolving healthcare landscape.

## 2 Methods

The study used a cross-sectional survey design with New Zealand registered pharmacists. Ethical approval was granted from the University of Auckland Human Participants Ethics Committee (Reference number 22499).

### 2.1 Study participants

Several sources were used to recruit study participants. The Pharmacy Council of New Zealand (PCNZ) provided a list of emails of all registered NZ pharmacists who consented to be contacted for research purposes as part of their Annual Practicing Certificate (APC) (*N* = 3037). An email was sent to members on this list with information detailing the study aims and objectives and a link to the survey containing a Participant Information Sheet (PIS). The Pharmaceutical Society of New Zealand (PSNZ) agreed to advertise the study via their weekly email newsletter to members, which included a link to the online questionnaire. Social media platforms including LinkedIn, Twitter, and a community pharmacy chat group on Facebook (ca. 4500 members) were also used.

Inclusion criteria were all NZ registered pharmacists with a current APC for 2021. Exclusion criteria were intern pharmacists, pharmacy assistants, and/or pharmacy technicians; pharmacists who did not hold an APC; and registered pharmacists no longer working in the pharmacy profession.

### 2.2 Data collection

A questionnaire was developed to answer the study questions by adapting pre-existing surveys from the *Future of Work* literature (available as a supplementary file).^7^ The questions explored pharmacists' current views and experiences with STAARA technologies; which roles and processes in pharmacy were most likely to be affected by STAARA technologies now and in the future; career security concerns and career planning considering STAARA; and the influence of external factors, such as Covid-19, on STAARA and the future of work. The survey included items measuring agreement (‘strongly agree’ to ‘strongly disagree’), likelihood (‘extremely likely’ to ‘extremely unlikely’), and extent (‘to a very large extent’ to ‘to a very small extent’) of STAARA use; and ten optional, open-ended questions. Demographic data on age, gender, ethnicity, current and previous pharmacy work sector, District Health Board (DHB), and years of experience as a pharmacist were collected.

The questionnaire was piloted with four community pharmacists, one hospital pharmacist, and one academic pharmacist for clarity, structure, and flow of questions. Members from the Centre for Automation and Robotic Engineering Science (CARES) at the University of XXX were consulted on specific phrasing related to robotics and automation. Based on this feedback, ambiguous terminology was reworded, and repetitive or obscure questions were reformatted for clarity. Pilot survey results were excluded from the final dataset.

Qualtrics® (Qualtrics, Provo, UT, USA) was used to create and distribute the study questionnaire and manage data collection. The software afforded participants anonymity and allowed for convenient distribution of the questionnaire. The PIS was included as part of the survey. Consent was given via participants clicking a button to show they had understood and accepted participation in the study. Data were collected between 19th and 31st August 2021.

### 2.3 Thematic analysis

Thematic analysis was carried out through coding of free text responses with NVivo 12 software (QSR International, Melbourne, VIC, Australia). A general inductive approach was adopted.^24^ The questionnaire responses were initially coded by student researchers, with each team member thoroughly examining the text to highlight relevant quotes and phrases. This early coding formed the basis of a preliminary framework. However, to ensure the robustness of the analysis, the lead author independently reviewed all qualitative data. Following this review, the lead author collaborated with senior researchers to refine and develop the themes. This iterative process involved revisiting the coded data whenever new themes emerged to ensure comprehensive inclusion and accuracy. This collaborative and supervisory approach ensured that the final themes accurately reflected the data across all participant responses.

Content analysis was used to quantify and interpret the presence of certain words, phrases, concepts, or topics within the free-text responses.^25^ This was done to examine the occurrence of selected terms, which were then compiled into a table for comparison (Supplementary Table 1). By converting qualitative data into quantitative insights, content analysis allowed us to determine how prevalent certain perspectives were among the study participants and draw meaningful conclusions about the context and underlying messages. The theme of technology adoption in pharmacy has been framed through macro, *meso*, and micro lens.

### 2.4 Quantitative data analysis

Questionnaire data was directly exported from Qualtrics® into a Microsoft® Excel (Microsoft, Redmond, Washington, US) spreadsheet. Data were cleaned by removing incomplete responses that did not continue past the demographic section. For data analysis, ethnicity data were regrouped into European, Māori, Pacific Peoples, Asian, and Other; age groups were combined into ‘20–29’, ‘30–59’, and ‘≥60’ years according to previous work on generational attitudes to computers and associated technologies^26^; and years in practice were combined as ‘≤15’, ‘16–30’ and ‘≥31’. For work sector, ‘academia’, ‘industry’, ‘Primary Health Organization’ and ‘government’ were combined with ‘other’.

### 2.5 Statistical analysis

IBM SPSS software was used for statistical data analysis. Descriptive statistics were used to calculate the percentage means and standard deviations. A significance level of 0.05 was adopted for all statistical tests. Each independent variable was compared among five Likert item questions to investigate pharmacists' opinions, making this a one-way analysis.

This analysis used a linear regression, where both independent and dependent variables were measured on a 5-point Likert scale. The analysis focused on understanding the potential drivers for pharmacists' career planning. We measured pharmacists' career planning by employing two variables; namely focusing on pharmacists' readiness to learn new skills (*I am ready to learn new skills or re-train to remain employable in the future*) and whether they have already started this process (*I have begun career planning to factor in STAARA*). All items were measured on a five-point Likert scale (1 = Strongly Agree and 5 = Strongly Disagree) and as appropriate the scale was reversed (1 = Strongly Disagree 5 = Strongly Agree). In relation to this we examined the drivers, namely pharmacists' views of the current impact of STAARA on pharmacy work (*Which aspects of STAARA are most likely to impact pharmacy now?***)**, their attitude towards technology adoption (*I am an early adopter of technology*) and their concern over their job loss (*I am worried about STAARA putting pharmacy jobs at risk*). Pharmacists' view of the current impact of technology was assessed in all five components of STAARA. The answers were aggregated across these five areas by undertaking a reliability analysis, which demonstrated internal consistency with a Cronbach alpha of 0.73.

A two-step hierarchical regression was used to test the effect of the above factors on pharmacists' career planning. All models were significant at 0.01 significance level and explain between 10 and 13% of the variance in the dependent variable. In the first step, the key variables of interest were added, including pharmacists' view of the current impact of STAARA, their attitude towards technology adoption, and their concern over their job loss.

In the second step gender (with male as indicator category), ethnicity (with NZ European as indicator) age (49 and under as indicator), years in practice (20 years or less as indicator) and job title (owner, manager, and pharmacists as indicator categories) where added and regressed to pharmacists' career planning.

## 3 Results

From the 3037 emails sent, 185 responses were received giving an initial response rate 6.09%. Of these, 8 were incomplete and were excluded from analysis. This resulted in 177 responses that met the inclusion criteria for analysis yielding a final response rate of 5.82%.

### 3.1 Pharmacist demographics

Over half the respondents were female (*n* = 105, 59.3%), between the ages of 30 and 59 (*n* = 92, 52.0%), European (*n* = 120, 67.8%), working in the community pharmacy sector (*n* = 116, 65.5%), and in practice for 15 years or less (*n* = 74, 41.8%) (Table 1). Pharmacists worked within all 20 District Health Board (DHB) catchments in NZ, with the majority in the Auckland region (*n* = 55,31.1%).

**Table 1:** Participant demographics (*n* = 177).

**Sex**		**Previous work sector/s**	
Female	105 (59.3%)	Community Pharmacy	156 (50.8%)
Male	72 (40.7%)	Hospital Pharmacy	75 (24.4%)
**Ethnicity**		Academia	20 (6.5%)
European	120 (67.8%)	Primary Healthcare Organization	18 (5.9%)
Asian	43 (24.3%)	Industry	17 (5.5%)
Māori	4 (2.3%)	Government (Medsafe, MOH)	11 (3.6%)
Pacific Peoples	2 (1.1%)	Other	10 (3.3%)
Other	8 (4.5%)		
**Age group (years)**		**District Health Board catchment**	
20–24	14 (7.9%)	Auckland	24 (13.6%)
25–29	30 (16.9%	Counties Manukau	
30–39	30 (16.9%)	Waitemata	16 (9.0%)
40–49	25 (14.1%)	Waikato	15 (8.5%)
50–59	37 (20.9%)	Canterbury	15 (8.5%)
60–69	36 (20.3%)	Capital and Coast	12 (6.8%)
≥70	5 (2.8%)	Southern	12 (6.8%)
**Years in practice**		Bay of Plenty	10 (5.6%)
<1–15	74 (41.8%)	MidCentral	10 (5.6%)
16–30	49 (27.7%)	Hutt Valley	7 (3.9%)
≥31	54 (30.5%)	Nelson-Marlborough	7 (3.9%)
**Current work sector**		Northland	7 (3.9%)
Community Pharmacist	66 (37.3%)	Hawke's Bay	6 (3.4%)
Hospital Pharmacist	41 (23.2%)	Taranaki	5 (2.8%)
Community Pharmacy (Owner)	29 (16.3%)	Lakes	4 (2.3%)
Community Pharmacy (Manager)	21 (11.9%)	South Canterbury	3 (1.7%)
Other	20 (11.3%)	Wairarapa	3 (1.7%)
		West Coast	3 (1.7%)
		Whanganui	3 (1.7%)
		Tairawhiti	1 (0.6%)
		Other	4 (2.3%)

**Number of pharmacists (%).**

### 3.2 Qualitative results

Two primary themes were developed from the qualitative data analysis: 1) factors affecting technology adoption at the macro, *meso* and micro levels, and 2) career impacts and implications. Together with associated subthemes, these themes provide a comprehensive insight into how pharmacists are navigating and adapting to the changes and challenges presented by a rapidly advancing technological world. Theme development is supported by quotes with additional quotes presented in the Supplementary Table 2.

### 3.3 Theme 1: Factors affecting technology adoption at the macro, meso and micro levels

Enablers and barriers of technology adoption fell under macro: global/country level (*COVID-19 pandemic's influence on work efficiency*) and policy, regulations and systems level (*absence of government support*, *absence of a clear regulatory environment*, and *fragmented health IT infrastructure*); *meso*: organizational level (*fragmented organizational infrastructure; patient safety requirements; workforce attitudes*); and micro: individual level influences.

### 3.4 Macro: Global/country level

• **Pandemic's influence on work efficiency**

The COVID-19 pandemic accelerated technology adoption in the pharmacy sector serving as a catalyst for automation and efficient systems and urging “*pharmacy to be more open to STAARA.”* This was reflected by the sudden necessity of remote work and need for telehealth capabilities.

Given the increase in workload and the pressure of staffing shortages, robots and other forms of automation were considered useful during high-volume periods, offering the added advantage of not being susceptible to illness.

*“Covid times have seen greater fluctuations in volume of work for pharmacies. In instances of very high volume, automation and robotics is very useful. Covid may have pushed more business owners in this direction. Robots also can't catch Covid and are thus available to work even if other staff members are compromised due to exposure.”*

Electronic prescriptions became the norm during this time and facilitated email as the primary route of prescription delivery to limit physical interactions. This was seen as a game-changer as it not only streamlined operations but also aided in pandemic-specific challenges, such as the need for contactless payment technology and signature-exempt prescriptions. It was also thought to highlight the flexible and resilient nature of pharmacists.

*“The use of ePrescriptions and contactless methods of receiving prescriptions has highlighted that pharmacy is ready to adopt change and is flexible and resilient. We are problem solvers and have used STAARA successfully to alter our services during the pandemic.”*

While the pandemic *“increased demand and pushed for innovation,”* it also disrupted supply chains, causing delays in the delivery of new technology and put financial constraints on pharmacies, *“restricting the income for businesses,”* which, in turn, limited their capacity to invest in new technologies.

### 3.5 Macro: Policy, regulation, system level

• **Absence of government support**

Affordability was frequently cited as a significant barrier to technology adoption. Some believed that the current state of pharmacies, particularly corporate stores, and government hospitals, couldn't afford new technology like STAARA as automation would require substantial validation. Financial help from District Health Boards (DHBs) was found to be lacking.

*“I work for a public hospital, so I don't think we will be early adopters of any technology related processes because of the lack of funding in general. We are very slow to change.”*

*“No financial help from DHB or our many rest home owners. All seem to demand but not pay for anything.”*

• **Absence of a clear regulatory environment**

There was a strong desire for more comprehensive policies around STAARA, and even an overhaul of the personnel in decision-making positions by governing bodies like the Ministry of Health (MoH), DHBs, and other organizations.

Many believed that aligning new technologies with existing legislation and regulations presented a formidable challenge. A combination of outdated regulations, bureaucratic inertia, and lack of understanding from key decision-makers and regulatory bodies was believed to constrain the potential for innovation and progress in the pharmacy sector. Existing legislative and regulatory frameworks were seen as out-of-date and obstructive, and described as moving at a *“glacial speed,”* affecting the rate of adoption.

*“Pharmacists have always been ready adopters of new technology, however our progress has continued to be held back by blinkered legislators, bureaucrats and the pay-masters within DHBs.”*

“*It will be very hard to get legislation and regulations updated as fast as technology and STAARA is.”*

• **Fragmented health IT infrastructure**

Furthermore, the fragmentation of New Zealand's health IT infrastructure was also seen as a key factor in technology adoption needing careful regulatory scrutiny.

*“Given the fragmentation and lack of fitness for purpose of much of NZ's health IT infrastructure, this is going to take a long time and require a paradigm shift in thinking and purchasing towards vertically integrated systems.”*

*“Having adequate hardware infrastructure to support any changes has been a major issue in the past and I don't see it changing within our DHB anytime soon.”*

### 3.6 Meso: Organizational level

• **Fragmented organizational infrastructure**

Most pharmacists highlighted the importance of user-friendly and efficient systems. The greatest emphasis was on accuracy, cost, physical size, ease of use, ability to integrate, and time-saving features. Other factors included *“better connections with the community”, “timely person to person access when errors or clarification”* were needed, and cheap maintenance to lighten workload.

Many pharmacists believed that current technology was not adequately reliable, and electronic prescriptions introduced *“inconsistencies and issues that need clarification/human management,”* requiring extra work to resolve these problems and leading to what some believed was a degradation in the *“quality of prescribing.”*

*“Unfortunately, because Prescriber software and NZePS are so poor, a lot of additional work has been created ‘fixing’ errors all day.”*

The increased workload was due to errors imported into pharmacy systems, as well as the lack of a common data platform across different systems, compounded by the perception that technology was not adequately tailored to the healthcare setting, making information gathering difficult and leading to an increase in medication errors and workarounds by clinical staff.

*“There were so many systems that did not communicate to each other, thus gathering information became difficult and slowed down the care I can provide.”*

Others pointed out the logistical challenges of unreliable technology causing delays and frustrations when upgrades didn't go through., stating that technology *“only works when the electricity is on,”* and when systems fail, *“it is a nightmare.”*

For technologies to be well-integrated - not only within the pharmacy setting but also extending to prescribers and broader healthcare systems - both pharmacist staff and employers expressed a need to be involved in the development of STAARA to ensure they were *“fit for purpose”* and to bridge the gap between innovation and real-world application.

*“The technologists seem to think that their systems are awesome. It often seems as though they have never actually looked at what happens in the real world.”*

However, there was a concern regarding the lack of time or motivation to trial new systems, especially with healthcare systems running at peak levels.

*“Very few have the time or motivation to take on extra work (potentially unpaid) to trial such ventures unless it's already outlined and proven to work.”*

Concerns were also raised about the practical implications of technology integration, especially in areas prone to natural disasters or cyber-attacks.

*“In case of something like hacking, can you imagine the chaos, damage, and serious consequences it could have.”*

Many also believed that larger urban pharmacies would be better placed to adopt technologies due to their capacity to invest. As a result, smaller and rural pharmacies who were unlikely to the same ability, would compete in a skewed marketplace and exacerbating existing inequities within the healthcare system. Technologies were seen as becoming “*a privilege for few.”* In other cases, pharmacies endeavored to adopt technological solutions, even in the face of fiscal limitations.

• **Patient safety requirements**

Patient safety was considered paramount with the introduction of new technology. There was a consensus that smart technology could improve by enhancing medication adherence and reducing medication error, but rigorous monitoring and data collection was still needed to ensure safety and accuracy.

*“Hopefully smart technology can kick in to help improve client compliance - e.g. see if doses have been taken or missed. Can track this somehow/set up better reminder systems / ensure things have been taken appropriately to help to keep peoples medication management in the home independent.”*

Technology additionally served as a supplementary support for human operators, with some envisioning the potential for a synergistic partnership to prevent patient harm.

*“the errors made by machines are very different to the ones made by humans. By having both involved in a dispensing process, the likelihood of errors is much lower.”*

• **Workforce attitudes**

One of the key factors for successful technology adoption was the willingness of both staff and employers to adapt to change. Despite the occasional *“resistance to change”* from some, most pharmacists believed that an engaged and willing workforce that actively participates in technology development and who were prepared to share their experiences, as crucial.

“*Willingness of early adopters to share their success and failures.”*

While some pharmacists identified as *“early adopters”* who were eager to integrate new technologies, others were cautious and slow to change, preferring to wait and see how technologies performed in other settings before adopting them.

*“Quite limited so far. I prefer other pharmacies to try it out before I adopt a new system. I do not want to repeat their mistakes if possible.”*

*“Mixed acceptance from the team - on the spectrum of early adopters to ‘hope it doesn't happen until after I retire.’”*

### 3.7 Micro: Individual level attitudes

From an individual perspective, the integration of technologies in the pharmacy sector was considered by many pharmacists as an *“exciting time”* with just under half of the pharmacists in the survey considering themselves as active users of technology. Most users had prior experience with dispensing and packing robots, regarding robotics and automation as particularly useful tools that could make their jobs easier.

*“I see that STAARA may make my life easier, I can source information quicker, I can lose the more boring parts of my job sooner - I see it as positive as long as I can control it rather than it controlling me.”*

However, the rapid advancement of technology in the healthcare sector also caused stress and anxiety, especially among older users who struggled to adapt, and introduced new complexities that were difficult to manage.

*“Stress and anxiety with rapid change and pharmacies have varied technology skills and other stressors at the time with staff / business as usual concerns.”*

To successfully integrate new technology into healthcare settings, in-depth training and support were considered essential for understanding how to use certain technologies in their practices. This included training for direct and end-users of the systems.

*“In-depth training and support systems for pharmacists and pharmacy staff are needed to understand how to use equipment and what it means with regards to changes in their roles.”*

### 3.8 Theme 2: Career impacts and implications

The challenges and opportunities that pharmacists saw technology play in their work can be summarized by three main subthemes: *broadening and evolving professional capacities*, *fear of replacement - balancing technology with job security*, and *embracing change through adaptation and upskilling.*

• **Broadening and evolving professional capacities**

The integration of technology into pharmacy practice was overwhelmingly viewed as a positive evolution, opening doors for pharmacists to expand their roles and refine their career paths. Most pharmacists believed that advancements in technology would broaden the variety of roles with emphasis placed on the increasing accessibility of pharmacists to patients, enabling roles in education, teaching, and professional services.

Pharmacists were already seeing traditional tasks like *“counting, snipping, and packaging,”* which consume a considerable amount of time, increasingly automated. This shift away from manual dispensing was seen as liberating and an opportunity to *“steer pharmacy to a more clinical role*” and for pharmacists to reassert their roles as vital members of the healthcare ecosystem, focusing more on *“patient care, optimization rather than dispensary processes.”*

Some pharmacists recognized that their roles would transition to “*knowledge navigators,”* given the rise of self-diagnosis through wearable devices and online information. Additionally, many envisaged a future where *“advice and consultation will occur through video conferencing or telehealth,”* emphasizing the potential for remote care.

Automation and technology were also seen as enablers, freeing pharmacists to apply their clinical expertise in more direct and meaningful ways. Many were looking forward to focusing on more services, like vaccinations, lifestyle coaching, and fast point of care testing. A few believed they would be able to offer individualized, biological-based treatments informed by advancements like DNA technology and contribute to health screening purposes in future. Tech-savvy pharmacists saw the opportunity to *“enhance the informatics stream”* of their careers, while others foresaw a more significant role for pharmacists in multi-disciplinary teams, emphasizing the need for an interdisciplinary approach to healthcare.

*“I hope that the future of work will be a close collaboration between IT experts and pharmacists, nurses and doctors.”*

The sentiment was that *“the possibilities are endless”* and there was excitement for future opportunities that not only increased professional enjoyment but also created a better system for integrated patient care.

• **Fear of replacement - balancing technology with job security**

The increasing uptake of technologies brought concerns for some regarding job security and the future of traditional roles in pharmacy. Some feared potential job losses, especially for dispensary technicians, while many felt that technology wouldn't eliminate pharmacist positions but rather reshape them.

*“I don't think the demand for pharmacists will decrease, I think the role will shift towards a more practitioner type role, doing people-facing jobs that machines and AI can't do.”*

While some acknowledged that there may be a *“reduction of available jobs leading to more competition and reduced prospects with lower remuneration,”* others remained skeptical that technology, within its current capacity, would supplant employment roles.

“I've noticed more technology often means more people in the department—just different roles.”

*“We use so many robots in our pharmacy and we still have four pharmacists and two technicians as there are many other responsibilities a pharmacist holds.”*

Some concerns were raised about maintaining the balance of technology and human interaction, stressing that a holistic approach is crucial in healthcare. The prevailing view held that technology would augment rather than replace the pharmacy workforce, with the majority acknowledging that STAARA could significantly improve efficiency and quality control in healthcare, but should *“never sacrifice the human touch and the human intellect”*. The fundamental duties of pharmacists, particularly in complex areas like clinical decision-making and patient counselling, and providing emotional support were likely to remain unaffected and secure.

*“There is no way to replace pharmacist checking of prescriber intentions, as algorithms cannot totally account for the wide range of variables influencing prescribing.”*

*“Hopefully I will have retired before being completely replaced by a cyborg, but otherwise not too concerned. Currently, STAARA isn't looking likes replacing the ‘soft’ skills - relational and communicative aspects of the profession - any time soon. AI systems such as Google Duplex may be able to arrange a hair appointment, but not discuss choices of anticoagulant therapy.”*

*“I think we need to remember health is not just a science but an art also. This is where the human touch can never be replaced by a machine.”*

Some pharmacists were concerned that an over-reliance on automation could devalue their profession, making patient care less personalized, affecting patient outcomes negatively.

*“Peoples mental health would be adversely affected because of less human interaction.”*

Additionally, pharmacists indicated that the public might not have been fully comfortable yet with the idea of being served solely by machines, especially in contexts requiring empathy, ethical decision-making, and complex situational awareness.

*“I know our customers like a human to tell them about their medicines.”*

• **Embracing change through adaptation and upskilling**

Despite some uncertainty about their future, there was an air of resilience and adaptability among pharmacy professionals. Many argued for taking a proactive approach, where the profession evolves to incorporate what technology can offer, rather than trying to fit new tech into old models of service.

*“Future career prospects will be based on how fit for purpose I am to practice in the newer changing environment. Consequently my learning goals will need to take account of any changes that may be presenting themselves. My role today is light years away from what it was when I qualified 38 years ago.”*

The majority of pharmacists expressed the need to diversify their skills or engage in upskilling and training initiatives to maintain their relevance in an increasingly technology-driven landscape. A variety of training mechanisms were mentioned, including webinars, online courses, and specialized postgraduate training in informatics. Many highlighted the potential for their roles to include more specialized skills, such as coding in languages like R, Python, and SQL, which was seen as crucial to operate various technologies.

*“Continuing to go for trainings about new aspects of dispensary systems, learning about machine-made blister packs, attending webinars about future of pharmacy.”*

*“I think we need to adapt to the times but it's not going to be an easy transition. We'll need to learn new skills such as how to operate robots, programming, etc.”*

Some pharmacists contemplated pursuing advanced studies to qualify as pharmacist prescribers or shifting their focus to healthcare team collaboration and system informatics. Meanwhile, a few pondered exiting the profession for various reasons. Conversely, others viewed emerging avenues, such as participation in vaccination initiatives, as a natural extension of their professional capabilities.

*“Upskilling. Learning new skills - vaccinating. Applying for jobs that utilize my skills but not necessarily in a pharmacist role.”*

### 3.9 Quantitative and descriptive analysis

Supplementary Table 3 presents the Pearson correlation coefficients, significance levels, and descriptive statistics for survey items related to technology adoption, concerns about STAARA, and career planning among pharmacists, highlighting significant relationships. When predicting pharmacists' readiness to learn new skills, Model 1 (Table 2) shows that being an early adopter of technology (b = 0.22(0.08), *p* < .01) positively influences (i.e. increases) pharmacists' readiness to learn new skills. In contrast, their concern about job losses due to STARRA (b = −0.15(0.06), *p* < .05) has a negative effect on (i.e. reduces) their readiness, whereas their view on various aspects of the technology had no significant effect (b = 0.13(0.11), *p* > .1) on their readiness. In Model 2, when adding demographic and job-related effects, being an early adopter and their concern remain significant predictors – positive and negative, respectively –at 0.05 significance level, however, none of the demographic and job-related factors have a significant effect on pharmacists' readiness for learning new skills.

**Table 2:** Two-step hierarchical regression.

		Technology adoption: I am ready to learn new skills or re-train to remain employable in the future						Career planning: I have begun career planning to factor in STAARA				
		Unstandardized Coefficients	Std. Error	Beta	t	Sig.		Unstandardized Coefficients	Std. Error	Beta	t	Sig.
Model 1 (F(3.155) = 6.58, p < .01; Adj R2 = 0.1)	(Constant)	3.45	0.53		6.57	0.00	Model 1 (F(3.155) = 7.64, p < .01; Adj R2 = 0.11)	0.75	0.71		1.05	0.29
	I am an early adopter of technology	0.22	0.08	0.23	2.85	0.00		0.29	0.10	0.22	2.84	0.01
	I am worried about STAARA putting pharmacy jobs at risk	−0.15	0.06	−0.18	−2.37	0.02		−0.12	0.08	−0.11	−1.45	0.15
	Impact of STAARA	0.13	0.11	0.09	1.10	0.27		0.39	0.16	0.19	2.52	0.01
Model 2 (F(10.148) = 3.45, p < .01; Adj R2 = 0.13)	(Constant)	3.27	0.59		5.57	0.00	Model 2 (F(10.148) = 3.34, p < .01; Adj R2 = 0.13)	0.46	0.81		0.58	0.57
	I am an early adopter of technology	0.16	0.08	0.17	2.06	0.04		0.20	0.11	0.15	1.88	0.06
	I am worried about STAARA putting pharmacy jobs at risk	−0.14	0.06	−0.17	−2.21	0.03		−0.09	0.09	−0.08	−1.08	0.28
	Impact of STAARA	0.15	0.12	0.10	1.31	0.19		0.45	0.16	0.22	2.81	0.01
	Gender (Male)	−0.05	0.16	−0.03	−0.32	0.75		0.33	0.21	0.13	1.57	0.12
	Ethnicity (NZ European)	−0.15	0.16	−0.08	−0.94	0.35		0.31	0.22	0.12	1.41	0.16
	Age (49 years or younger)	0.36	0.27	0.19	1.34	0.18		0.68	0.37	0.27	1.86	0.06
	Years in practice (20 or less)	0.07	0.27	0.04	0.26	0.79		−0.07	0.37	−0.03	−0.20	0.84
	Position(Owner)	0.25	0.28	0.10	0.88	0.38		−0.18	0.39	−0.05	−0.46	0.65
	Position(Manager)	0.05	0.29	0.02	0.16	0.88		−0.63	0.40	−0.16	−1.59	0.11
	Position(Pharmacist)	0.18	0.23	0.09	0.77	0.44		−0.34	0.31	−0.13	−1.10	0.27

When predicting whether they have started their career planning for STAARA, Model 1 shows that similarly to predicting readiness, being an early adopter of technology (b = 0.29(0.1), *p* < .01) significantly and positively affects pharmacists' career planning, whereas their concern about job loss has no significant effect (b = −12(0.08), *p* > .1). However, their views of various aspects of STAARA (b = 0.39(0.16), *p* < .05) positively influences their career planning. Model 2 shows that only the age of pharmacist respondents (being 49 or younger) had a (b = 0.68(0.37), *p* < .1) positive effect on career planning, whereas other demographic and job-related factors did not have a statistically significant effect on pharmacist career planning for STAARA.

A summary of findings based on the thematic and statistical analyses is presented in Table 3.

**Table 3:** Enablers and barriers highlighted at macro-meso-micro-levels in relation to technology adoption or career planning.

	**Technology adoption**		**Career planning**	
	**Enablers**	**Barriers**	**Enablers**	**Barriers**
**Macro-level:** global (pandemic) or country-level factors (policy, regulations, systems)	Better work efficiency	Greater workload/stress Lack of government/ financial support Lack of clear regulations Fragmented health IT infrastructure	Broadening and evolving professional capacities	
**Meso-level:** organizational level factors		Fragmented organizational infrastructure Workforce attitudes		
**Micro-level:** individual level factors	*Being an early adopter*	*Fear of replacement due to STAARA*	*Being an early adopter* *Impact of STAARA* Embracing change through adaptation and upskilling	Fear of replacement due to STAARA

Note: findings presented in italics are based on the statistical analysis, all else are based on thematic analysis.

## 4 Discussion

The study aimed to explore the views and experiences of New Zealand pharmacists regarding the impact of STAARA technologies on the pharmacy profession - concerns about career security, the adoption of new technologies, and how their roles might evolve - including the effects of the COVID-19 pandemic on technology implementation and future work dynamics.

### 4.1 Participant demographics as they relate to the findings

Compared with the national average pharmacy workforce in New Zealand,^27^ the ethnicity profile of our survey respondents showed a higher proportion of Europeans (67.8% vs 47.4%), and a lower proportion of Asians (24.3% vs 36.0%), and Māori and Pacific Peoples representation aligning well. Gender distribution also differed slightly, with a higher proportion of male pharmacists (40.7% vs 33%) in our study. Workforce representation was lower for community pharmacists (50.8% vs 75.9%) and higher for hospital pharmacists (24.4% vs 13.8%) compared to the national proportions. These demographic factors did not significantly influence career planning in our study.

In New Zealand, the median age of a practicing pharmacist is 37.5 years. Pharmacists aged between 25 and 39 years, comprised 33.9% of our study cohort. Our study finds that while age may correlate with a propensity for technological adoption, the actual willingness and ability to embrace new tools and practices varied among individuals within all age groups. Existing research suggests that a younger demographic is generally more open to adapting to technological changes than their counterparts over the age of 55^2^^,^^28^; however, such findings are not universally consistent, and can be attributed to small sample sizes or a high proportion of younger participants in studies, skewing the outcomes. The idea that older individuals are universally resistant to change due to entrenched habits and beliefs requires a nuanced understanding. Whether a pharmacist is early in their career or has decades of experience, familiarity with traditional practices can foster skepticism towards innovative healthcare technologies.

### 4.2 Comparison with the literature

Integrating technology into pharmacy practice presents a range of challenges, as highlighted across different levels in our study. Globally, the COVID-19 pandemic served as a significant catalyst for the rapid adoption of automation and artificial intelligence, propelling virtual transactions such as telemedicine into the mainstream.^29^ The relevance of these technologies is well-documented in recent pharmacy literature, with a bibliometric review identifying “telemedicine” as one of the most prevalent MeSH terms.^30^ However, while the shift to remote work became more common in many sectors,^29^ our study participants highlighted the vulnerability within the New Zealand pharmacy sector in that the existing model of care did not incorporate telehealth, preventing pharmacists from leveraging this technology during the pandemic. Furthermore, while automation was employed to manage increased workloads and facilitate electronic prescription transfers, systemic vulnerabilities such as disruptions in supply chains and financial constraints posed challenges in technological investments. Several key institutional barriers significantly hindered the adoption of technology in the pharmacy sector in our study during this time. These included the absence of robust government support for tele-pharmacy policies, inadequate regulatory frameworks, and fragmented healthcare IT systems. Infrastructure and technical challenges are common barriers to digital health technology adoption, encompassing issues such as inadequate network infrastructures, slow connectivity, limited access to electricity, and restricted capacity for technology integration.^31^ Post-pandemic, several governments failed to provide a sufficient evaluation of pharmaceutical care eHealth services, resulting in the absence of a suitable remuneration system, and with significant delays in the development of comprehensive legal frameworks and policies needed to support eHealth in primary care.^23^ Inadequate funding and a lack of communication infrastructure necessary for timely scaling up affected both hospitals and community pharmacies,^23^ slowing the uptake of technology during the pandemic and highlighting the importance for substantial governmental and regulatory support to ensure continuity, quality and efficiency of care.

At the *meso* level, our study identified organizational change as a crucial driver for technology adoption. Pharmacists acknowledged the potential of these technologies to boost operational efficiency, promoting work approaches that are smarter, not harder. For successful implementation, pharmacists require technologies that are accurate, user-friendly, and efficient - characteristics that are essential for minimizing errors, enhancing reliability, and integrating seamlessly with existing systems. Additionally, the importance of patient safety was emphasized, often considered more critical than merely improving efficiency. The adoption of digital health technologies is significantly influenced by healthcare professionals' willingness to embrace these tools, spurred by their belief in the technology's capacity to enhance job performance.^31^ These design requirements are supported by other studies, underscoring their significance not only for optimal technology performance but also for effective data management.^31^

Another crucial factor for technology adoption is the readiness of the workforce to embrace change and willingly share their experiences. Early technology adopters were viewed as ‘agents of change’ whose responsibility it was to share their successes and failures with others who were slower to adoption. Whole of workforce resistance to change has hindered the adoption of innovative services and roles within pharmacy^32^^,^^33^ and is often fueled by challenges such as difficulty in understanding new technologies, fear of reduced human interaction, and technophobia. These barriers are further compounded by factors such as education levels and professional experience, which can significantly influence the acceptance and effective use of digital tools.^31^ Data from this review^31^ underscores the importance of tailored educational and training programs that meet the specific needs of healthcare professionals. Our study's findings corroborate the need for such programs, which, when combined with high-quality, real-time technical support and coaching, are crucial for technology adoption. These initiatives not only facilitate a smoother transition and integration of new technologies but also help in alleviating fears and skepticism, ultimately leading to improved efficiency and reduced internal conflicts during system adoption.

At the individual level, pharmacists in our study perceived technology adoption as an opportunity to streamline their workflow, allowing more time for rewarding tasks. They were optimistic about the potential of STAARA technologies to expand their roles as knowledge navigators, providing a stronger clinical focus and enhanced opportunities for interdisciplinary collaboration in healthcare. This is supported by literature that advocates for pharmacists to evolve and expand their professional roles to increase their capacity for innovative service provision.^34^^,^^35^ To realize this vision, there is an urgent call within the pharmacy profession to rapidly embrace digital technologies and cultivate digital competencies across the profession.^36^

The readiness of pharmacists to learn new skills, as observed in our study, was significantly influenced by their attitudes towards technology adoption. Early adopters, recognizing the potential benefits such as career adaptation, job security, and increased adaptability, were more inclined to acquire new skills or re-train in a rapidly evolving professional landscape. In contrast, concerns about job losses due to technology negatively impacted some pharmacists' willingness to learn new skills, leading them to consider transitioning to roles perceived as less vulnerable to technological disruption or even exiting the profession—sometimes through retirement. These effects appeared independent of demographic or job-related factors.

Previous research has highlighted ‘automation anxiety’ among pharmacists, a concern that can be alleviated by embracing change, adapting to new roles, and upskilling.^37^ Despite this, there has been a notable deficiency in career planning strategies to effectively manage this transition and address the underlying anxieties associated with automation. Our study found that early adopters of technology, and those who perceived a significant potential impact of STAARA on their careers currently, were more proactive in planning their careers. This included seeking additional training and upskilling in relevant areas, or even altering their career paths to harness the expected changes brought about by these technologies. Interestingly, concerns about potential job losses due to STAARA did not significantly influence career planning among pharmacists. However, those younger than 50 were more likely to engage in career planning activities, likely because they have a longer career horizon within which to navigate the changes in the job market.

Sofia Hernnäs’^38^ research on the impact of automation across various sectors provides insights into how pharmacists are responding to technological advancements. She suggests that while digitization may replace some traditional roles, it also significantly augments non-routine, cognitive tasks, which are abundant in healthcare settings. Additionally, the evolution of technology often leads to the creation of new tasks and roles, offering pharmacists further opportunities to innovate and expand their professional capabilities in a digitized environment.

Hernnäs' findings also illuminate how adaptation and skill enhancement have historically helped workers across industries navigate the challenges of automation.^38^ This aligns closely with our observations that pharmacists who proactively embrace STAARA technologies are more engaged in upskilling and career planning. Such proactive behavior is crucial, as it reflects broader labor market dynamics where adaptability to technological changes often determines job security and career progression. Furthermore, understanding automation anxiety through a comparative industry-wide lens reinforces the importance of strategies like training and role adaptation in managing these anxieties. By embracing these approaches, pharmacists can mitigate their fears and harness the potential of new technologies to expand their roles as knowledge navigators, thereby enhancing their clinical focus and interdisciplinary collaboration. This perspective underscores the importance of viewing technological disruptions not merely as challenges but as significant opportunities for professional growth and innovation in pharmacy practice, encouraging a forward-thinking approach amidst evolving healthcare landscapes.

Furthermore, themes from our qualitative data emphasized the importance of balancing technology with the human aspect of pharmacy. This balance is vital as pharmacists adapt to evolving roles where technology augments but does not replace human interactions. By emphasizing technology as a tool to enhance pharmacists' capabilities, we ensure that while they leverage new digital solutions for efficiency and accuracy, the core of pharmacy practice—personal patient care and nuanced clinical judgment—remains intact. This integration speaks to the broader narrative of modern healthcare, where technology serves as an enabler of better patient outcomes^39^^,^^40^ through its support of the pharmacist's expanding role,^41^^,^^42^ rather than as a replacement for the personal touch that is fundamental to effective healthcare delivery.

### 4.3 Implications for education and practice

The implications for education and practice in pharmacy are multifaceted, especially considering the accelerated adoption of STAARA technologies driven by the COVID-19 pandemic. This shift has not only altered the “future of work” but also necessitated a robust technological infrastructure to support remote working and studying, which are likely to continue evolving.^29^ As the delivery of pharmacy services undergoes strategic reassessment, our study underscores a significant transition towards more dynamic roles that extend beyond traditional dispensing. These include clinical decision-making, patient counselling, and the administration of expanded services such as health screenings, immunizations, smoking cessation, and needle exchange programs.^10^^,^^41^

To effectively navigate these changes, pharmacists must master digital tools and data management to enhance service delivery and improve patient interactions. The integration of technology in pharmacy practice is essential not just for operational efficiency but also for elevating the quality of care, positioning pharmacists as vital components of the modern healthcare ecosystem.

Consequently, there is a pressing need for structured digital technology training across all levels of pharmaceutical education—from pharmacy students to experienced practitioners.^43^ Implementing tailored frameworks like the NHS Digital Capabilities Framework,^44^^,^^45^ adapted specifically for the New Zealand context, is crucial. This training will equip pharmacists not only to adapt to new technologies but also to thrive, ensuring they remain at the forefront of a rapidly evolving digital healthcare landscape.

### 4.4 Limitations

This study has several limitations. The survey was launched two days after Auckland entered an Alert Level 4 lockdown on August 17th, 2021. This was followed by a nationwide lockdown on August 20th, which lasted until August 31st for most regions. Community pharmacists were heavily involved in the COVID-19 vaccination rollout, leading to high levels of exhaustion and stress. We received feedback from a couple of pharmacists expressing their frustration with the timing of the survey, prompting us to only send one email invitation out, and closing the survey on August 31st to avoid further burdening the respondents. This is likely to have skewed the demographic profile of the respondents, which does not fully mirror the national averages, potentially affecting the generalizability of the findings across different demographic groups.

As the study relies on self-reported data, this can introduce the social desirability bias, where participants might overestimate their adaptation to or enthusiasm for technology. The cross-sectional design of the study also limits our ability to draw causal inferences or observe changes in attitudes over time. Finally, while thematic analysis was employed to interpret the qualitative data, it is important to note that these themes are subjective and based on the interpretations of the research team.

### 4.5 Future research

While this study has provided valuable insights into pharmacists' perspectives on STAARA technologies, it has primarily considered them in isolation. Pharmacists operate within broader healthcare ecosystems that include a range of professionals such as pharmacy assistants and technicians. Future studies should expand to include these other stakeholders to obtain a holistic view of how technology impacts entire pharmacy organizations and the interactions within.

Further studies are needed to explore the longitudinal effects of these technologies on pharmacist roles, job satisfaction, and patient outcomes. It is essential to investigate how different demographics within the pharmacy workforce adapt to technological changes, particularly focusing on the interplay between age, experience, and adaptability to innovation. Additionally, in-depth interview-based studies would be beneficial to capture the nuanced perspectives of health policymakers, funders, and planners regarding the future of work in pharmacy. These discussions could reveal important policy and funding gaps that influence technology adoption and integration.

Another critical area for future research is the digital competency of pharmacists, particularly in New Zealand, where this competency is essential for the successful implementation of new technologies. Investigating the extent of digital skills among pharmacists will help in identifying educational deficiencies and informing the development of targeted training programs. This approach will ensure that pharmacists are not only prepared to embrace new technologies but are also equipped to excel in an increasingly digital healthcare landscape. Such research will guide policy-making and educational strategies, ensuring they are aligned with the evolving needs of the healthcare sector.

## 5 Conclusion

This study explored pharmacists' views on the “future of work” amidst the 4th Industrial Revolution. Identifying as an early adopter of technology was found to be a predictor of readiness to learn new skills and engage in proactive career planning. Our research, highlighting a gap in existing literature, revolved around two main themes: technology adoption and career planning. Using a macro/*meso*/micro analytical approach, our study revealed how the COVID-19 pandemic, regulatory frameworks, and policy support are shaping the future of work, alongside an organizational willingness to embrace change, fear of job replacement, and the need for pharmacists to broaden their professional skills.

The global relevance of these findings suggests the need for broader international research to compare these insights with global expectations. Understanding the perspectives of broader pharmacy team members, including assistants and technicians, is crucial for optimizing service delivery. Additionally, the role of policymakers and the integration of technology-focused curricula in pharmacy education are critical for equipping future pharmacists to succeed in a digitally evolving healthcare landscape. Addressing these diverse aspects will help the pharmacy sector effectively meet the challenges and opportunities of the digital era, fostering robust and innovative practice models for the future.

## CRediT authorship contribution statement

**Nataly Martini:** Supervision, Project administration, Methodology, Formal analysis, Conceptualization, Writing – review & editing, Writing – original draft. **Laszlo Sajtos:** Formal analysis, Writing – review & editing. **Lynette Idio:** Data curation, Writing – original draft. **Manvinder Kaur:** Data curation, Writing – original draft. **Nicole Sweeney:** Data curation, Writing – original draft. **Carrie Zhang:** Data curation, Writing – original draft. **Shane Scahill:** Methodology, Conceptualization, Writing – review & editing.

## Declaration of competing interest

The authors declare that they have no known competing financial interests or personal relationships that could have appeared to influence the work reported in this paper.

## Appendix A Supplementary data

Supplementary material Image 1
