# Supplementary material for: The future of pharmacy work: How pharmacists are adapting to and preparing for technology infusion
Source: Explor Res Clin Soc Pharm. 2024 Jul 5;15:100472. doi: 10.1016/j.rcsop.2024.100472 (PMC11300926; doi:10.1016/j.rcsop.2024.100472)
Supplement: Supplementary file 1 — Supplementary material [file mmc1.docx]

Supplementary Table 1: Content analysis of various impacts and responses to STAARA on the future of pharmacy work

| **Survey item** | **Details** |
| --- | --- |
| Personal levels of technology engagement (n=171) | Active user: 45; little/none: 105 |
| Current pharmacy workplace engagement with STAARA (n=171) | Active: 50 (mostly robotics: 34); little/none: 101 |
| Pharmacy staff most affected by automation (n=156) | All staff (especially dispensing staff): 81; mostly technicians: 52; no-one: 4 |
| Processes most affected by automation (n=153) | Dispensing (incl counting, pouring) : 112; blister packing: 63; interaction/checking: 19 |
| What will the future of pharmacy work look like in 10 years due to STAARA? (n=155) | More clinical consultations: 70; more services: 11; telehealth: 7; better clinical decision support systems: 25; will be retired: 15; no change: 9 |
| Effect of STAARA on career prospects (n=152) | Role changes (more clinical/patient focused): 62; minimal/no effect: 26; fewer jobs: 15; retired/leaving the profession: 22 |
| Career planning in light of STAARA (n=141) | Learning additional skills: 75; considering changing roles: 16; leaving the profession/retiring: 19; no planning: 31 |
| Impact of COVID-19 on pharmacy work related to STAARA (n=145) | Minimal/no impact: 23, increased opportunity: 96, more work stress: 17 |

|  |  |
| --- | --- |
|  |  |
|  |  |
|  |  |
|  |  |
|  |  |
|  |  |
|  |  |

Supplementary Table 2: Additional quotes from the thematic analysis

| **Theme 1: Technology adoption** | | |
| --- | --- | --- |
| **Macro:**  **Global/country level** | **Pandemic’s influence on work efficiency** | *"Covid-19 has definitely identified the favourable aspects of STAARA in terms of what can be done if people are accessing work remotely."*  *“We also realised how vulnerable our workforce is and that working from home doesn’t feature in our current model of care. We don’t have telehealth set up so couldn’t take advantage of that.”*  *“It* [Covid-19] *has made STAARA far more important, as it has shown how much more flexible it makes our service.”* |
| **Macro:**  **Policy, regulations, systems level** | **Absence of government support** | *“Opportunities are there but these people keep trying to force a change on us while refusing to do any financial modelling to help us understand what their plan is. It can only end in disaster if they have their way.”* |
|  | **Absence of a clear regulatory environment** | *"The elephant in the room is the glacial speed of change in our regulatory environment.* *We still exist on 1970s legislation which is hampering any advancement in many areas. Our Medsafe auditors are stuck in the prehistoric mindset of writing things in books, rather than such technology as computer recording of Controlled Drugs etc.."*  *"STAARA could significantly improve patient safety, but I'm not sure if much more can be achieved under current legislation and Pharmac policies."* |
|  | **Fragmented health IT infrastructure** | *“High level of engagement but due to poor investment in NZ Health IT and few options for the NZ market due to our small population our experience with STAARA has been very poor and has led to an increase in medication errors, work arounds by clinical staff as it is often not fit for a variety of different clinical settings and is implemented by non-clinical staff with expertise in IT but don't understand health, how clinical staff work and use IT systems that are inflexible and hard coded.”* |
| **Meso: Organizational level** | **Fragmented organizational infrastructure** | *"I would prefer to use more of our software in the pharmacy and make technology do more routine tasks to work smarter, not harder."*  *"Doctors seem to be so busy they are not interested in using new online technologies such as e-prescribing unless it is very simple and no problems with it."*  *“Given the professions we interact with to carry out our duties I would say that it is almost an unmitigated failure. E.g. WRT EScripts for instance, we now import errors directly into our pharmacy systems that the human (i.e. pharmacist or tech) used to weed out doing data entry. Prescribers, without exception are wholly incompetent at data entry. I am tired of having to do more work to correct their failures within our system.”* |
|  | **Patient safety requirements** | *“With any new technology, it is vital to collect data and monitor feedbacks closely to ensure safety.”*  *“Focus on medication and patient safety rather than just on increasing efficiency.”* |
|  | **Workforce attitudes** | *"A willing workforce that sees benefit in the change and not more mindless administration for middle management."*  *“We work now in complex, high pressured environments- if we can use algorithms and computerised technology to aid us and minimise errors, I'm all for it.”* |
| **Micro:**  **Individual Level** |  | *"I should mean I get to do more of what I love and less of what I don't."*    “*It will free me up to do the things I enjoy. I won’t have to count and pour ever again. I will be free to discuss important things with my customers. I will be able to finish work early and go and enjoy my time off because the robots will have it all taken care of.”*  *"Use some algorithms and wish there were more available—it would free up a lot of time to spend on things that can't be computerized.”* |
| **Theme 2: Career impacts and implications** | | |
|  | **Broadening and evolving professional capacities** | *"It will broaden the variety of roles available in the future and help innovate our profession. Professional services will play a key role due to our training and accessibility to patients."*  *“We will need to be knowledge navigators as people will have access to self-diagnosis (wearable devices for health) and use the internet for treatment options - but that is fraught with inaccuracies / marketing etc so we will need to have extensive and well-honed skills in literature searching (complete) and critical appraisal to avoid providing biased and untrue information. Artificial intelligence is further away, so we still need to be able to apply the results from mega-data / algorithms to an individual.”* |
|  | **Fear of replacement - balancing technology with job security** | *"if we can pivot ourselves towards this new future then we will not have to fear being replaced...We need to start making parts of our services which only we are skilled to do available online."*  *“STAARA at this stage has only limited application and leads to huge complications in changing human-run tasks to technology-run tasks. Every time we put new technology in to replace people there become a new set of problems. These problems are usually as a result of the unforeseen new situations caused by the technology itself and the human part of the job becomes service to the problems that machines throw up. If anything there is a decrease in real productivity and more job dissatisfaction as staff play second fiddle to the technology. This means the level of human pharmaceutical care suffers. Believe me when I say that in all my years of pharmaceutical practice that I have seen these trends. Our faith in the technological fix is often misplaced!”*  *"Greater automation may improve workflow, but not human interaction that is vital in the delivery of patient-focused healthcare."*  *"So much of what I do is based around the acute awareness of my patients' situations, not just their health."*  *"The healthcare is for the people and optimizing their healthcare needs to have people still ultimately in charge of how that is provided."*  *"You can have all the technology in the world, but the patient is human; technology can't make them take the medicine.”* |
|  | **Embracing change through adaptation and upskilling** | *"I think that if we keep doing what we are doing now we will be replaced and fight to survive...We don’t want to be the video store when the signs of DVD, blue-ray, and Netflix are there that we need to adapt, innovate and change or die."*  *“The profession needs to change and adopt what STAARA can bring, not try and stay in the same environment and try and adapt what STAARA can bring to an old model of service delivery.”*  *“Making sure I stay up to date with the extra training that Pharmacists can do to ensure that I am an asset to the pharmacies that I work at.”*  *“Keeping up to date with technology news, and becoming more familiar with the more technical and software parts of the multi tablet counter and sachet robot.”*  *“Pharmacists new to the profession need to be aware that they need to continue to upskill - and be cognisant of the need to have a wide field of knowledge, and that includes a significant amount of STAARA ...”* |

|  |  | Item 1 | Item 2 | Item 3 | Item 4 | Item 5 | Min | Max | Mean | Std. Deviation |
| --- | --- | --- | --- | --- | --- | --- | --- | --- | --- | --- |
| I am an early adopter of technology (1) | Pearson Correlation | 1 | -.187^*^ | .228^**^ | .279^**^ | .287^**^ | 1.00 | 5.00 | 3.74 | 0.98 |
|  | Sig. (2-tailed) |  | 0.018 | 0.004 | 0.000 | 0.000 |  |  |  |  |
| I am worried about STAARA putting pharmacy jobs at risk (2) | Pearson Correlation | -.187^*^ | 1 | -0.011 | -.225^**^ | -0.155 | 1.00 | 5.00 | 2.82 | 1.16 |
|  | Sig. (2-tailed) | 0.018 |  | 0.887 | 0.004 | 0.052 |  |  |  |  |
| Impact of STAARA (3) | Pearson Correlation | .228^**^ | -0.011 | 1 | 0.139 | .246^**^ | 1.60 | 5.00 | 3.95 | 0.64 |
|  | Sig. (2-tailed) | 0.004 | 0.887 |  | 0.082 | 0.002 |  |  |  |  |
| I am ready to learn new skills or re-train to remain employable in the future (4) | Pearson Correlation | .279^**^ | -.225^**^ | 0.139 | 1 | .508^**^ | 1.00 | 5.00 | 4.35 | 0.94 |
|  | Sig. (2-tailed) | 0.000 | 0.004 | 0.082 |  | 0.000 |  |  |  |  |
| I have begun career planning to factor in STAARA (5) | Pearson Correlation | .287^**^ | -0.155 | .246^**^ | .508^**^ | 1 | 1.00 | 5.00 | 3.05 | 1.29 |
|  | Sig. (2-tailed) | 0.000 | 0.052 | 0.002 | 0.000 |  |  |  |  |  |

Supplementary Table 3: Descriptive statistics for survey items

**The future of work – pharmacists’ perceptions of smart technology, automation, artificial intelligence, robotics and algorithms (STAARA) Questionnaire**

This survey is divided into three sections:

● Section A requests demographic information.

● Sections B - F asks about your current experiences, then perceptions of the impact of STAARA.

● Section G provides an opportunity for you to provide comments on the future of work in pharmacy as it relates to STAARA.

**Section A: Demographic Information**

Q1. Gender:

▢ Male

▢ Female

▢ Gender diverse

▢ Prefer not to state

Q2. Which ethnic group do you belong to?

▢ New Zealand European

▢ Māori

▢ Samoan

▢ Cook Island Māori

▢ Niuean

▢ Chinese

▢ Indian

▢ Other (please specify)

Q3. Age group:

▢ 20 - 24 years

▢ 25 - 29 years

▢ 30 - 39 years

▢ 40 - 49 years

▢ 50 - 59 years

▢ 60 - 69 years

▢ 70 years and above

Q4. Number of years in practice:

▢ <1

▢ 1 - 5

▢ 6 - 10

▢ 11 - 15

▢ 16 - 20

▢ 21 - 25

▢ 26 - 30

▢ 31 or more

Q5. Which of the following describes the area you MOSTLY work in?

▢ Community Pharmacy (Owner)

▢ Community Pharmacy (Manager)

▢ Community Pharmacy (Pharmacist)

▢ Hospital Pharmacy (Pharmacist)

▢ Academia

▢ Industry

▢ Primary Healthcare Organisation

▢ Government (i.e Medsafe, Ministry of Health)

▢ Other (please specify)

Q6. Which areas have you previously worked in? Tick all that apply.

▢ Community Pharmacy

▢ Hospital Pharmacy

▢ Academia

▢ Industry

▢ Primary Healthcare Organisation

▢ Government (i.e Medsafe, Ministry of Health)

▢ Other (please specify)

Q7. Which DHB in New Zealand do you work under? Please specify:

**Section B: Your views on STAARA**

Q1. Please indicate your answer to each question by selecting one of the options below

Strongly agree (1), Somewhat agree (2), Neither agree nor disagree (3), Somewhat

disagree (4), Strongly disagree (5)

1. I am an early adopter of technology
2. I am worried about STAARA putting pharmacy jobs at risk
3. I am an advocate for the adoption of STAARA-type technologies now and into the future

Q2. How would you describe your personal levels of engagement with STAARA?

Q3. How would you describe your pharmacy workplace and engagement with STAARA?

**Section C: Impact of STAARA on workload in pharmacy**

Please indicate your answer to each question by selecting one of the options below Extremely likely, Somewhat likely, Neither likely nor unlikely, Somewhat unlikely, Extremely unlikely

Q1. Which aspects of STAARA are most likely to impact pharmacy **now**?

- Smart technology
- Automation
- Artificial Intelligence
- Robotics
- Algorithms

Q2. Which aspects of STAARA are most likely to impact pharmacy **in the future**?

- Smart technology
- Automation
- Artificial Intelligence
- Robotics
- Algorithms

Q3. When thinking about roles in pharmacy, who is affected by automation?

Q4. When thinking about processes in pharmacy, which processes are most affected by automation?

Q5. Describe what you think your work will look like in 10 years as a result of STAARA

**Section D: Career security concerns**

Q1. Please indicate your answer to each question by selecting one of the options below

To a very large extent (1), To a large extent (2), To a moderate extent (3), To a small

extent (4), To a very small extent (5)

1. To what extent is the pharmacy sector at risk of automation?
2. To what extent do you think that technology may replace people in pharmacy?
3. To what extent do you fear that technology may replace your role in pharmacy?

Q2. Describe how STAARA may affect your future career prospects

**Section E: Career planning as a result of STAARA**

Q1. Please indicate your answer to each question by selecting one of the options below

Strongly agree (1), Somewhat agree (2), Neither agree nor disagree (3), Somewhat

disagree (4), Strongly disagree (5)

1. I am ready to learn new skills or re-train to remain employable in the future
2. I have begun career planning to factor in STAARA

Q2. What sorts of things are you thinking about doing as you plan your career in light of STAARA?

**Section F: Factors impacting on future of work in pharmacy in relation to STAARA**

Q1. How do you think COVID-19 has impacted pharmacy as it relates to STAARA?

Q2. What do you see as important factors that impact on the future of work when it comes to STAARA?

**Section G: Additional comments**

Please include any further comments about the future of work in pharmacy as it relates to STAARA that may not have been covered by your previous answers
